# The Impact of Inclusive Leadership on Employees’ Innovative Behaviors: The Mediation of Psychological Capital

**DOI:** 10.3389/fpsyg.2019.01803

**Published:** 2019-08-06

**Authors:** Yang-Chun Fang, Jia-Yan Chen, Mei-Jie Wang, Chao-Ying Chen

**Affiliations:** ^1^Global Institute for Zhejiang Merchants Development, Zhejiang University of Technology, Hangzhou, China; ^2^The School of Management, Zhejiang University of Technology, Hangzhou, China; ^3^Zhejiang ChangZheng Vocational & Technical College, Hangzhou, China

**Keywords:** new generation employees, inclusive leadership, psychological capital, innovative behavior, China

## Abstract

Employee innovation is the cornerstone of the organization, and the motivation for employee innovative behavior largely depends on the leadership style of the leader. With the economic development of society, the traditional authoritative style of leadership can no longer adapt to the psychological characteristics of employees, who use new-era work concepts, techniques, and social rules (hereafter, new generation employees). Inclusive leadership is based on the concept of “fully inclusive and equitable” in traditional Chinese culture, and it can adapt to the independent needs of new generation employees. At present, the research on the relationship between the traditional leadership style and employee innovative behavior is relatively extensive, but there is little research on the relationship between inclusive leadership style and employee innovative behavior, and this needs further exploration. This paper takes new generation employees as the sample and uses psychological capital as an intermediary variable to explore the influence of inclusive leadership style on the innovative behaviors of new generation employees. We found that inclusive leadership is significantly and positively related to new generation employees’ innovative behavior. Theoretical and practical implications are discussed.

## Introduction

Innovation drives enterprise development, and companies are relying more and more on their employees’ innovative contributions to maintain and improve their competitiveness (Odoardi et al., 2015). “New generation employees” have gradually become the new enterprise workforce. We mean to expand the traditional meaning of the term “new generation employees” to fit today’s realistic workplace situations. We refer to the term “workers of new era,” which describes those workers who complete their jobs with new-age concepts, techniques, and social rules in mind. Accordingly, workers’ composition is not necessarily related to the sole factor of demographic age. For example, an older employee may be a new generation employee because of his/her renewed working philosophy learned through continuous education and higher educational degrees. Such workers may not be well led by leaders using traditional leadership models, which is why we conduct the present study. New generation employees have different working values from the previous generation’s traditional work values (Hou et al., 2014), challenging traditional leadership and governance methods. Increasing numbers of scholars have focused their research on issues such as high turnover rate and low organizational commitment (Cheng and Lin, 2017).

The effects of certain leadership styles may have different consequences for different generations in the workplace (Al-Asfour et al., 2014). To date, few studies have explored the suitability of different types of leadership styles for generational working groups and how to stimulate their innovative behaviors. Leadership, as an important organizational scenario variable, has an important impact on employees’ innovative behaviors (Zubair et al., 2015; Liu et al., 2017). Among leadership styles, the inclusive leadership style emphasizes being people-oriented (Liu et al., 2017), fairness, and justice (Liu et al., 2016), which might be suitable for the new generation workers mentioned above. Therefore, this paper introduces the inclusive leadership style into the research on the innovative behavior of new generation employees.

Furthermore, new generation working styles require new governance approaches, which depart from concrete, tangible, pre-defined rules to more diverse, flexible, and intangible motivators (Chen and Zhou, 2018). For example, new generation employees may contribute to innovative working behaviors by using self-stimulated psychological motivators (e.g., need for achievement) rather than organizational demands and orders (McClelland et al., 1976), especially in an innovative or entrepreneurial context (Hansemark, 1998). In addition, new generation employees may have a different state of positive psychological power that is beneficial to the generation of innovative behaviors (Staples, 2014). The effects of certain leadership styles on new generation employees’ innovative behaviors should be influenced by collective psychological state more than is seen in a traditional workplace. To examine this, we used psychological capital as an intermediary variable to explore how inclusive leadership impacts the innovative behavior of new generation employees. This paper enriches the research on inclusive leadership and innovative behavior among new generation employees, and it provides theoretical guidance for management to strengthen new generation employees’ advantages and increase their innovative behavior.

## Theory and Hypothesis

### Inclusive Leadership

Inclusion is written into the UN Millennium Development Goals and is a historical feature of Chinese civilization (Yuan, 2007). Inclusiveness is a traditional virtue of the Chinese nation. The meaning of “All rivers run into the sea” and “Wide hearts embrace all” both encapsulate the meaning of inclusiveness.

The inclusive leadership style was initially studied in the field of Western education. People of different races and abilities should be educated inclusively. Ryan believed that inclusive leadership in education requires an equal collective leadership process and defined inclusive leadership in education as the presence of a learning leader (Ryan, 2007). For the first time, Nembhard and Edmondson (2006) proposed inclusive leadership in the field of management, which comprises the speech and behavioral performance of leaders in encouraging their subordinates to work and contribute. Hollander (2009), emphasized the perceived role of employees in leadership and defined this relationship as an interdependent one that is both win-win and has a shared vision. Based on Hollander’s research, Carmeli, and Reiter believed that inclusive leadership can be judged from the interaction between leaders and employees and that inclusive leadership is open, effective, and accessible in the process of communication with employees (Cameli et al., 2010). Hirak et al. (2010) used a large hospital as a study sample and found that inclusive leadership had a significant positive impact on subordinates’ psychological security. Wiebren studied the concept and measurement of inclusion. They believed that inclusion should be composed of two components: belonging and authenticity (Jansen et al., 2014). Inclusion is defined as the sense of belonging and security from the team. Suk posited inclusive leadership as an open, effective, and accessible method of leadership that is positively correlated with employee performance (Choi et al., 2016).

Chinese scholars started late in the study of inclusive leadership, but many explorations are still ongoing. Fang (2014) believed that inclusive leaders pay great attention to the relationship between leaders and followers, combining the characteristics of transformational leadership and transactional leadership, taking advantage of authentic leadership and shared leadership style. Liu et al. (2016) proposed that inclusive leadership pursues the principle of being people-oriented, insists on equal treatment toward subordinates’ attitudes, believes in the role of organizational cohesion, and takes its own efforts as an example. Liu et al. (2017) proposed that inclusive leadership adheres to being people-oriented, advocates individuality and difference, attaches importance to leadership-employee interaction, and is good at listening to subordinates’ opinions and contributions.

Based on the literature about inclusive leadership, this paper integrated the concept of “inclusiveness” from Chinese traditional culture into that of inclusive leadership. The concept of inclusiveness in the West was mainly derived from the ideas of democracy and justice. In Chinese culture, “inclusiveness” is more about the “tolerance and greatness” of the mind and moral cultivation. The inclusive leadership that integrates Chinese traditional culture emphasizes equal opportunity and fair distribution, in line with higher psychological pursuits and the respected needs of the new generation employees, is a new type of democratic leadership. Inclusive leaders are able to treat employees with recognition, respect, and tolerance, listen to and recognize the opinions and contributions of subordinates (Sharifirad, 2013), and promote their work performance (Choi et al., 2015). At the same time, inclusive leaders pay attention to employee training, give employees fair treatment, and drive business success (Yuan, 2007). Inclusive leaders can help each other in interacting with their subordinates (Nishii and Mayer, 2009). It is this “relational leadership” that interacts with leaders and employees (Cameli et al., 2010) and is responsible for the final outcome. Inclusive leadership is an embodiment of openness and fairness (Zhu and Wang, 2011).

In the design of their questionnaire scale, Nembhard and Edmondson (2006) divided the inclusive leadership into two dimensions: the leadership’s “invitation” and “appreciation” of the team members. Hollander (2009) developed an Inclusive Leadership Scale that includes “support-recognition,” “communication-action-fairness,” and “self-interest-disrespect” through in-depth interviews.

Based on the previous literature and empirical research, we introduced the concepts of recognition, encouragement, and inclusiveness into the leadership practices of leaders in the new era. (1) Leaders should listen to the opinions of employees, attach importance to encouragement of employees, and show their recognition when employees make achievements; (2) Leaders should respect and treat employees fairly. That is, the leaders can treat employees fairly, justly respect the employees’ suggestions, and let employees work more to receive more; (3) Leaders should rationally understand employees and tolerate their failures. That is, when employees make mistakes, leaders can rationally tolerate and understand them.

### Employees’ Innovative Behavior

The concept of innovative behavior began in the 1970s. Innovative behaviors consist of three levels: organizational, team, and individual innovative behavior. This paper studies the individual innovative behavior of enterprise employees. Amabile (1988) believed that employee creativity is a novel, potentially valuable idea or thing that employees can generate, which can encourage companies to survive, grow, and thrive in fierce competition. Woodman et al. (1993) believed that the ideas generated during the innovation process can be novel or have been applied by others. Zhou and George (2001) believed that individual innovative behavior not only refers to the birth of an innovative concept but also its promotion and implementation.

Woodman et al. (1993) believed that employee innovative behavior includes the process of generating creative ideas and successful implementation. Scott and Bruce (1994) believed that innovation was divided into three phases: (1) the establishment of problems and the creation of solutions; (2) seeking support for their ideas; and (3) generating innovative standards or models that can be spread, mass-produced, and then used in large quantities. Kleysen and Street (2001) grouped individual innovative behaviors into five stages: finding opportunities, generating ideas, forming surveys, supporting, and applying.

Scholars in China has also conducted research on employee innovative behavior. Liu and Shi (2009) and Han and Yang (2011) defined employee innovative behavior as the creation and implementation of novel and practical methods when employees conduct related activities in the enterprise. Li (2017) believed that employee innovative behavior refers to the process by which employees discover problems, generate innovative ideas, promote and implement them throughout the life of the organization. Based on the questionnaires of Scott and Bruce, this paper divided innovative behavior into two dimensions: innovation outcomes and innovative thinking. Innovative thinking refers to new ideas arising from employees’ work or production process, and innovation outcomes refers to the effects of implementing new ideas into the work and production processes.

### New Generation Employees

New generation employees are more active in their work and have stronger willingness and ability to learn at work than previous generations (Li and Xu, 2013). New generation employees, who have strong creative ability, are not willing to be bound by the rules. They prefer fair, just, democratic, and simple working relationships. New generation employees tend to be more achievement-oriented and self-oriented, and they tend to focus on equality and disregard authority. New generation employees also have characteristics of working values such as pursuing a balance between work and life (Li and Hou, 2012). These characteristics have led to lower job satisfaction and organizational commitment and higher turnover and occupational mobility rates for new generation employees (Twenge et al., 2010). New generation employees are more committed to organizational fairness and justice, emphasizing equal relations with leaders. They are more eager to be recognized and respected, which is challenging to the traditional methods of human resources management.

### Inclusive Leadership and Employees’ Innovative Behavior

Cultivating innovative behavior is one of the most important leadership functions of today’s organizations (Pundt, 2015; Hakimian et al., 2016). Employees’ ability to innovate is significantly related to leadership style (Lee and Chang, 2006). For example, a leader with humor can encourage expression of creative ideas as an innovative behavior (Pundt, 2015). In addition, transformational leaders are good at stimulating employees to innovate by engaging their intelligence and motivation (Zhang and Zhou, 2013).

Like those positive leadership styles, inclusive leadership also has beneficial effects from the Chinese cultural perspective. Employees are more innovative when working at a higher level of engagement because they think their efforts have won the leaders’ accolades (Abdullan et al., 2015). Employees’ innovative behavior is also influenced by leadership support. Employees are more adventurous and innovative when the leaders support them (George and Zhou, 2007).

Fang (Fang, 2014) put forward the “fault-tolerant concept” of inclusive leadership in the Chinese situation and analyzed its positive influence on employees’ self-efficacy with concrete examples. Liu et al. (2017) found that inclusive leadership positively predicts teams’ mental models, and teams’ reflection moderates the relationship between them. More directly, Jing (2015) found that inclusive leadership has a significant positive impact on employees’ creativity in China. Jin et al. (2017) suggested that the more inclusive employees feel, the more likely they are to improve their performance. Randel et al. (2017) conceptually defined inclusive leadership as a group of positive leadership behaviors that can help team members feel the sense of belonging to the team and maintain their uniqueness within the team. Therefore, leaders with an inclusive leadership style have more positive expectations and tolerance for employees, which allows employees to feel more support from the leaders and then generate more ideas (Zhu and Wang, 2011). For new generation employees who generally have more creative ideas but have views that contrast with the traditional leadership style, such inclusiveness incorporating encouragement and tolerance is more effective. Hence, we propose the following hypotheses:

Hypothesis 1: Inclusive leadership has a positive impact on new generation employees’ innovative behavior in China.
Hypothesis 1.1: Inclusive leadership style has a positive impact on new generation employees’ innovation outcomes.
Hypothesis 1.1.1: Leaders’ encouragement and recognition of new generation employees have a positive impact on their innovation outcomes;
Hypothesis 1.1.2: Leaders’ respect and fair treatment of new generation employees have a positive impact on their innovation outcomes;
Hypothesis 1.1.3: Leaders’ rational understanding and tolerance of new generation employees’ failures have a positive impact on their innovation outcomes.
Hypothesis 1.2: Inclusive leadership has a positive impact on new generation employees’ innovative thinking.
Hypothesis 1.2.1: Leaders’ encouragement and recognition of new generation employees have a positive impact on their innovative thinking;
Hypothesis 1.2.2: Leaders’ respect and fair treatment of new generation employees have a positive impact on their innovative thinking;
Hypothesis 1.2.3: Leaders’ rational understanding and tolerance of new generation employees’ failures have a positive impact on their innovative thinking.

### Psychological Capital

Psychological capital reflects an optimistic attitude toward work and life (Chen and Lim, 2012). The concept first appeared in the related fields of economics, investment, and sociology, and it emphasizes individuals’ positive psychological resources and motivational tendency (Luthans et al., 2007; Zhong, 2007).

The discussion of psychological capital can be divided into two categories: those based on economics and psychology or organizational behavior. The concept of psychological capital based on economics emphasizes the relatively stable psychological tendencies or characteristics that individuals develop in their early years of life (Goldsmith et al., 1997, 1998). The concept of psychology in terms of organizational behavior emphasizes the characteristics of psychological capital that can be measured, developed indefinitely, and managed (Zhong, 2007). The academic community has not yet reached a consensus about the constituent dimensions of psychological capital. The most widely used structure by the academic community is Luthans’ construct, which consists of the following four dimensions. Self-efficacy means having the confidence to undertake challenging tasks and try to complete (Luthans and Youssef, 2007); hope is mainly composed of three conceptual foundations: cravings, pathways, and goals (Luthans et al., 2007); optimism refers to positive emotions or motivations associated with good outcomes (Luthans, 2002); and resilience refers to seeking positive changes in setbacks such as conflicts and failures (Luthans, 2002). The research on psychological capital-related variables has mostly focused on employees’ job performance, job satisfaction, employee work happiness, turnover intention, and work slack (Zhong, 2007). Psychological capital as a positive psychological factor impacts employees’ behavior, and their level of psychological capital can predict employees’ positive or negative behavior to a certain extent.

Inclusive leadership positively impacts employee self-efficacy (Fang, 2014). When leaders pay attention to their employees’ needs, motivations, and communication, the employees become more optimistic and confident in their work. Inclusive leadership behaviors facilitate group members’ perceptions of inclusion, which in turn lead to member work group identification, psychological empowerment, and behavioral outcomes (creativity, job performance, and reduced turnover) in the pursuit of group goals (Randel et al., 2017). Most have agreed that the employees’ mood is affected by leaders’ recognition and appreciation (Nembhard and Edmondson, 2006). Positive support from leaders enhances employees’ psychological capital (Şahin et al., 2014). When leaders show an open, accessible attitude toward employees and communicate effectively with employees, their confidence and hopes are higher (Edmondson, 1996). Awareness of psychological safety among employees is positively correlated with inclusiveness among leaders (Hirak et al., 2010). Inclusive leaders are more willing to communicate with and give feedback to their subordinates (Edmondson, 1999), and they also pay more attention to employees’ participation (Bass and Bass, 2009). Thus, inclusive leadership can actively promote employees’ psychological capital through strengthened self-efficacy and other dimensions (Fang, 2014). Employees with higher levels of psychological capital can more often work with full enthusiasm (Edmondson, 1996, 1999; Luthans, 2002; Luthans et al., 2004; Bass and Bass, 2009; Şahin et al., 2014). Thus, inclusive leaders can enhance employees’ psychological capital to promote their innovative behavior by recognizing, encouraging, and respecting employees and tolerating employees’ failures. Therefore, this paper proposes Hypothesis 2.

Hypothesis 2: Inclusive leadership has a significant impact on employees’ psychological capital.

As an important psychological resource of organizational collectives, psychological capital could play a mediating role that transforms organizational-wide force/interventions (e.g., inclusive leadership) and organizational consequences. Psychological capital plays mediating roles between organizational innovation atmosphere and employees’ innovative behavior (Luthans and Youssef, 2007) and between transformative leadership and employees’ innovative behavior (Mao, 2008). Innovation atmosphere affects employees’ work behavior by affecting employees’ internal psychological state (Song et al., 2011). Thus, psychological capital as a collectively owned positive psychological state plays an important mediating role between inclusive leadership and employees’ innovative behavior (Dreu and West, 2001). Innovative behavior is not only stimulated by the objective external environment, but also motivated by subjective factors of individuals or collectives. Tierney and Farmer found that the sense of innovative self-efficacy had a significant positive impact on individual innovative behavior and that innovation self-efficacy can predict individual innovative behavior (Tierney and Farmer, 2004; Hassan et al., 2015). Psychological empowerment affects employees’ innovative behavior by influencing their internal and external motivation. Employees’ self-efficacy and ability to work stimulate their intrinsic motivation, and those with high self-efficacy show greater confidence and have more innovative behavior (Song et al., 2011). Employees tend to innovate actively if they perceive themselves in a fair, friendly and innovative organizational climate (Wang et al., 2013). In addition, highly activated positive emotions promote innovative behavior, while low-activated positive emotions are not related to innovative behavior (Pundt, 2015). Furthermore, when employees have hope in mind, they more easily predict their leader’s instructions or guidance for them (Byron, 2008) and may turn those into innovative thoughts and behaviors. Finally, when encountering challenging situations that require leadership effects to maintain employees’ resilience, successful resilience could lead employees to generate innovative thinking based on inclusive leaders’ words or helpful actions, as they can gain different experiences and reflections from challenging situations they would not encounter in routine practice. In summary, the four dimensions of psychological capital can each be examined as mediators that intervene on the influence of inclusive leadership on innovative behavior, leading to Hypothesis 3.

Hypothesis 3: Psychological capital plays a mediating role between inclusive leadership and employees’ innovative behavior.
Hypothesis 3.1: Psychological capital plays a mediating role between inclusive leadership and employees’ innovation outcomes.
Hypothesis 3.1.1: Psychological capital plays a mediating role between leaders’ encouragement and recognition of employees and employees’ innovation outcomes.
Hypothesis 3.1.2: Psychological capital plays a mediating role between leaders’ respect and fair treatment of employees and employees’ innovation outcomes.
Hypothesis 3.1.3: Psychological capital plays a mediating role between leaders’ rational understanding and tolerance of employees’ failures and employees’ innovation outcomes.
Hypothesis 3.2: Psychological capital plays a mediating role between inclusive leadership and employees’ innovative thinking.
Hypothesis 3.2.1: Psychological capital plays a mediating role between leaders’ encouragement and recognition of employees and employees’ innovative thinking.
Hypothesis 3.2.2: Psychological capital plays a mediating role between leaders’ respect and fair treatment of employees and employees’ innovative thinking.
Hypothesis 3.2.3: Psychological capital plays a mediating role between leaders’ rational understanding and tolerance of employees’ failures and employees’ innovative thinking.

## Research Methods

### Sample

We adopted a random questionnaire survey method, taking enterprise employees of Zhejiang as the research sample. We sent out a total of 372 questionnaires, and 351 valid ones were returned, resulting in a return rate of 94.35%. Among these people, 177 male and 174 female workers were questioned: 43.59% were aged less than 30 years; 19.37% were aged 30–39 years; 27.64% were aged 40–49 years; 7.69% were aged 50–59 years; and 1.71% were aged over 60 years. Most employees had a bachelor’s degree or above: 57.55% undergraduate, 10.54% master’s, and 3.70% doctorate. General staff accounted for 29.63% of the sample, followed by 19.09% middle layer managers, and 7.12% senior professional and technical personnel. Focusing on those who were aged above 40 years in the sample (i.e., those easily excluded from the group of new generation employees), *nearly 60% had earned an educational degree of bachelor’s or above, and over 20% of them used continuing education as a major tool to update work concepts and skills. In terms of job positions, over 20% of them were working as experts/professionals, and over 50% were managers. Both of those job types were characterized by high-level and fast-changing knowledge bases, and those sampled employees were therefore required to update themselves to fit new work concepts, models, and trends.*

### Research Tool

This article contains three scales. The Inclusive Leadership Scale was designed based on the pilot study and in-depth interviews. The Employee Innovative Behavior Scale and the Psychological Capital Scale were adopted from the questionnaires by Scott and Bruce (1994) and Luthans and Youssef (2007), but the expression was slightly modified according to employee characteristics. These three scales use Likert type 5-point scales. We listed all questionnaire items in the Appendix for readers’ reference. The details of scale development are explained below.

#### Reliability and Validity Tests

##### Inclusive Leadership Scale

Based on previous literature and previous surveys, we conducted questionnaire surveys and interviewed employees and leaders. The Inclusive Leadership Scale based on the questionnaire by Cameli et al. (2010) examines the concept of inclusive leadership style using structured interviews. First, factor analysis of the Inclusive Leadership Scale data was conducted based on 151 elements of scale data. The KMO (Kaiser-Meyer-Olkin) test and the Bartlett spherical test were performed to determine whether factor analysis could be performed. The KMO value was 0.936, and the value of the Bartlett spherical test was lower than 0.01, so factor analysis could be performed. The Inclusive Leadership Scale included three factors: the leaders’ encouragement and recognition of employees, the leaders’ respect and fair treatment of employees, and leaders’ rational understanding and tolerance of employees’ failures. These three factors accounted for 64.13% of the variation: 23.01, 20.79, and 20.33%, respectively. The factor loading of each item was higher than 0.528 and lower than 0.832. Then, we performed a reliability test using Cronbach’s alpha coefficient. The alpha coefficient of this scale was 0.930, which showed good reliability. A structural dimension test was conducted on the inclusive leadership style scale by confirmatory factor analysis based on 200 elements of scale data. We selected Chi-square/df, RMSEA, NFI, IFI, and CFI as evaluation criteria. The specific data are shown in Table 1. Chi-square/df was 2.83, RMSEA was 0.08, and NFI, IFI, and CFI were all above 0.9, indicating that the model fit the data well.

**Table 1:** Confirmatory factor analysis results of inclusive leadership style questionnaire (*N* = 200).

Scale	Chi-square/df	RMSEA	NFI	IFI	CFI
The inclusive leadership style scale	2.83	0.08	0.902	0.934	0.934

##### Psychological Capital Scale

The KMO value of the Psychological Capital Scale was 0.904, as obtained by analysis of the exploratory factor, and the results of the Bartlett spherical test were also significant at the 0.01 level. The Psychological Capital Scale includes four factors that accounted for a total of 63.89% of the variation: hope accounted for 20.33%, optimism accounted for 15.41%, toughness accounted for 15.16%, and self-efficacy accounted for 12.99%. The factor loading of each item was higher than 0.551 and lower than 0.868. The alpha coefficient of this scale was 0.888, showing good reliability.

##### Innovative Behavior Scale

The Innovative Behavior Scale uses Likert’s five-point scoring method. Factor analysis of this scale showed two factors that accounted for a total of 67.12% of the variation. The factors of employees’ innovation outcomes and their innovative thinking accounted for 34.03 and 33.09% of the variation, respectively. The factor loading of each item was higher than 0.690 and lower than 0.917. The alpha coefficient of this scale was 0.890, showing good reliability.

#### Descriptive Analysis

The results of the descriptive analysis are shown in Table 2. The dimensions of inclusive leadership were ordered according to average score, from high to low: F1 (Leaders’ encouragement and recognition of employees), F3 (Leaders’ rational understanding and tolerance of employees’ failures), and F2 (Leaders’ respect and fair treatment of employees). The average score on the dimension of employees’ psychological capital was 3.6, which was higher than the middle level. The average scores on the dimensions of employees’ innovation outcomes and innovative thinking were 3.59 and 3.78, respectively. It is of interest to determine how to transform employees’ innovative thinking into innovation outcomes, and this requires more attention during the process of team building.

**Table 2:** Results of descriptive and correlational analyses.

	Average	Standard deviation	N	F1	F2	F3	P	C1	C2
F1	3.81	0.62	351	1					
F2	3.58	0.83	351	0.643^∗∗^	1				
F3	3.60	0.77	351	0.656^∗∗^	0.746^∗∗^	1			
P	3.60	0.50	351	0.491^∗∗^	0.466^∗∗^	0.485^∗∗^	1		
C1	3.59	0.63	351	0.367^∗∗^	0.305^∗∗^	0.283^∗∗^	0.557^∗∗^	1	
C2	3.78	0.58	351	0.409^∗∗^	0.310^∗∗^	0.292^∗∗^	0.543^∗∗^	0.702^∗∗^	1

*F1 means leaders’ encouragement and recognition to employees; F2 means leaders’ respect and fair treatment of employees; F3 means leaders’ rational understanding and tolerance of employees’ failures; P means psychological capital; C1 means employees’ innovation outcomes; C2 means employees’ innovative thinking; ^∗∗^ means prominently positive relevance around 0.01; ^∗∗^*p* < 0.01.*

#### Correlational Analysis

The results of the correlational analysis in Table 2 show that the three dimensions of inclusive leadership also had significantly positive associations with psychological capital, innovation outcomes, and innovative thinking of employees. Psychological capital also had a significantly positive association with innovation outcomes and innovative thinking by employees. There are different correlation coefficients between the three dimensions of inclusive leadership and the psychological capital: the order from high to low is was F1, F3, and then F2. There were differences between the three dimensions of inclusive leadership and innovative behavior. The most strongly associated dimension of inclusive leadership with innovation outcomes and innovative thinking by employees was F1 (leaders’ encouragement and recognition of employees).

#### Mediation Analysis

To verify the influence of inclusive leadership on employees’ innovative behaviors, we used regression analysis. We also tested psychological capital as the mediating variables, and the results are shown in Table 3. Referring to Baron’s and Kenny’s (1986) methods of testing mediating mechanisms, mediating effects should obey the following conditions: independent variables significantly influence dependent variables; independent variables significantly affect mediating variables; and mediating variables significantly influence dependent variables. When the independent and mediating variables were substituted into the regression equation to explain the dependent variables at the same time, the effect of the mediating variables was significant, while the effect of the independent variables disappeared (all mediating effects) or weakened (partial mediating effects).

**Table 3:** The result of regression analysis.

	P	C1		C2	
	Model 1	Model 2	Model 3	Model 4	Model 5
Gender	–0.033	–0.026	–0.007	–0.138*	–0.122*
Age	0.108***	0.107*	0.047	0.064	0.012
Education	0.016	0.108***	0.099***	0.094**	0.086**
Working Time	–0.003	0.035	0.037	0.025	0.026
Job Position	0.004	0.009	0.007	0.020	0.019
F1	0.196***	0.252***	0.141*	0.298***	0.204***
F2	0.092*	0.115*	0.063	0.077	0.033
F3	0.140**	0.017	–0.062	0.013	–0.054
P			0.565***		0.481***
*R*^2^ (adj. *R*^2^)	0.357 (0.342)	0.238 (0.220)	0.369 (0.352)	0.267 (0.250)	0.378 (0.361)
F	23.700***	13.324***	22.130***	15.601***	22.978***

*F1 means leaders’ encouragement and recognition of employees; F2 means leaders’ respect and fair treatment of employees; F3 means leaders’ rational understanding and tolerance of employees’ failures; P means psychological capital; C1 means employees’ innovation outcomes; C2 means employees’ innovative thinking; ^∗^*p* < 0.05, ^∗∗^*p* < 0.01, ^∗∗∗^*p* < 0.001.*

The dependent variable in model 1 was employees’ psychological capital. The three dimensions of controlling variables and inclusive leadership collectively accounted for 34.2% of the variation in the dependent variable of employees’ psychological capital (*F* = 23.700, *p* < 0.001). Leaders’ encouragement and recognition of employees (F1, β = 0.196, *p* < 0.001), leaders’ respect and fair treatment of employees (F2, β = 0.092, *p* < 0.05), and leaders’ rational understanding and tolerance of employees’ failures (F3, β = 0.140, *p* < 0.01) were significantly associated with psychological capital. Therefore, Hypothesis 2 could be tested.

The dependent variable of models 2 and 3 was employees’ innovation outcomes. In model 2, the three dimensions of controlling variables and inclusive leadership collectively accounted for 22.0% of the variation in the dependent variables representing employees’ innovation outcome (*F* = 13.324, *p* < 0.001). Among these, leaders’ encouragement and recognition of employees (F1, β = 0.252, *p* < 0.001) and leaders’ respect and fair treatment of employees (F2, β = 0.115, *p* < 0.05) positively influenced employees‘ innovation outcomes, while leaders’ rational understanding and tolerance of employees’ failures (F3, β = 0.017, n.s.) could not significantly account for employees’ innovation outcomes. Therefore, Hypothesis 1.1.1 and 1.1.2 could be verified. In model 3, the controlling variables of inclusive leadership and psychological capital accounted for 35.2% of the variation in employees’ innovation outcomes (*F* = 22.130, *p* < 0.001). Leaders’ encouragement and recognition of employees (F1, β = 0.141, *p* < 0.05) significantly accounted for the employees’ innovation outcomes, while leaders’ respect and fair treatment of employees (F2, β = 0.063, n.s.) and leaders’ rational understanding and tolerance of employees’ failures (F3, β = -0.062, n.s.) could not significantly account for this. However, psychological capital could account for the employees’ innovation outcomes (β = 0.565, *p* < 0.001). F2 could significantly account for the psychological capital, but F1 had a weaker association. Therefore, leaders’ respect and fair treatment of employees influenced employees’ innovation outcomes through the mediating function of psychological capital, while leaders’ encouragement and recognition of employees partially mediated innovation outcomes through psychological capital. Therefore, Hypothesis 3.1.1 and 3.1.2 could be verified.

The dependent variable of models 4 and 5 was employees’ innovative thinking. In model 4, the three dimensions of controlling variables and inclusive leadership collectively accounted for 25.0% of the variation in the dependent variables representing employees’ innovative thinking (*F* = 15.601, *p* < 0.001). Among these, leaders’ encouragement and recognition of employees (F1, β = 0.298, *p* < 0.001) had a positive influence on employees’ innovative thinking, while leaders’ respect and fair treatment of employees (F2, β = 0.077, n.s.) and leaders’ rational understanding and tolerance of employees’ failures (F3, β = 0.013, n.s.) could not significantly account for employees’ innovative thinking. Therefore, Hypothesis 1.2.1 could be verified. In model 5, the controlling variables of inclusive leadership and psychological capital accounted for 36.1% of the variation in employees’ innovative thinking (*F* = 22.978, *p* < 0.001). Leaders’ encouragement and recognition of employees (F1, β = 0.204, *p* < 0.001) significantly influenced the employees’ innovative thinking, and psychological capital (β = 0.481, *p* < 0.001) significantly accounted for employees’ innovative thinking. F1’s explanatory ability for employees’ innovative thinking was weak. Therefore, leaders’ encouragement and recognition of employees partially mediated innovative thinking through psychological capital. Therefore, Hypothesis 3.2.1 could be verified.

The above analyses indicate that leaders’ respect and fair treatment of employees can influence the employees’ innovation outcomes through the mediation effect of psychological capital. Leaders’ encouragement and recognition of employees can influence employees’ innovation outcomes and innovative thinking through the mediation effect of psychological capital.

## Discussion and Implications

In today’s society, new generation employees have gradually become the main drivers of workplace and enterprise development. This group’s professional values and community characteristics are different from those of the traditional labor force, which leads to great pressure and challenges for many traditional human capital management and leadership styles. Psychological capital might be a good prescription for such stresses (Shabir et al., 2014). Based on the concept of inclusiveness in Chinese culture, we have explored the relationship between inclusive leadership and employee innovative behavior with psychological capital as a mediator. We conclude the following. First, inclusive leadership has a significant positive influence on new generation employees’ psychological capital and then innovative behaviors.

Second, we conclude that different dimensions of inclusive leadership have different influences on different dimension(s) of innovative behaviors. Among the three dimensions of inclusive leadership style, leaders’ encouragement and recognition of employees has a significant influence on new generation employees’ innovation outcomes and innovative thinking. Leaders’ respect and fair treatment of new generation employees has a significant influence on their innovation outcomes. Further, we provide detailed information about practical implications. Leaders adopting an inclusive style know more precisely how to generate different innovative results for new generation employees. Third, psychological capital plays a mediating role between leaders’ respect and fair treatment of employees’ innovation achievements. It also played a partial mediating role between leaders’ encouragement and recognition of employees’ innovation outcomes and innovative thinking. Such results add to the existing literature by clarifying psychological capital’s differentiated effects on different inclusive leadership-innovative behavior relationships. Fourth, different from the concept of fairness and justice in Western countries, this article integrates the traditional Chinese cultural aspect of “tolerance as a virtue” into the connotation of inclusive leadership style and emphasizes the concepts of tolerance and leniency. Combining the characteristics that differ between traditional employees and new generation employees, we used empirical research to verify how inclusive leadership style affects employees’ innovative behavior. The results promote the reasonable use of the inclusive leadership style, adaptation to the characteristics of new generation employees, and giving a full audience to new generation employees. The results ultimately provide a basis for boosting the innovative thinking and behavior of the new generation employees.

This paper provides theoretical bases and implications for future studies. It also supplements and expands the research on the relationship between inclusive leadership style and employees’ innovative behavior and provides new ideas for human resource management approaches among new generation employees.

Our results have practical significance for enterprise human resource management and the development of entrepreneurial leadership style in the new era. The results give leaders clear guidance regarding leadership style that can accommodate new generation employees’ characteristics, give full play to their advantages, and stimulate their innovative behaviors to facilitate enterprise development. In addition to caring about leadership style, leaders should simultaneously focus on cultivating employees’ psychological capital to create more advantages and value for enterprises resulting from innovation (Luthans and Youssef, 2007). Encouraging, recognizing, respecting, including, and giving fair treatment to employees are all excellent qualities of new-era leaders that can promote employee innovation.

This article has some deficiencies. The questionnaires about inclusive leadership, psychological capital, and innovative behavior were all filled in by the same person, which may cause homologous data errors. However, we have made some efforts to lower potential biases. Before we collected the data, we re-ordered the questionnaire items according to the independent and dependent variables, preventing the raters from guessing the causal relationships between variables; thus, we reduced the possible bias caused by having the same person provide answers for both independent and dependent variables (Kozlowski and Klein, 2000). After data collection, Harman’s test was conducted. The analytic results of the un-rotated factor solution showed that the variance of the principle component is 30%, which was less than 40%, and thus, no dominant single factor was extracted, indicating little potential for common variance bias. Additionally, our samples are all survey data from Zhejiang Province, China. This could raise some concern about generalizability. Although the currently surveyed city is representative of other similar cities in China, generalizability to other cities in other countries is indeed a common concern because the results might vary for cultural, institutional, or societal reasons. Follow-up studies could expand the scope of the survey and increase the number of samples to ensure the generalizability of the research conclusions.

## Data Availability

The datasets generated for this study are available on request to the corresponding author.

## Author Contributions

Y-CF conceived and designed the research and provided guidance throughout the entire research process. J-YC wrote and supplemented the English manuscript. M-JW and C-YC participated in the data processing and writing of the Chinese Papers.

## Conflict of Interest Statement

The authors declare that the research was conducted in the absence of any commercial or financial relationships that could be construed as a potential conflict of interest.

## Appendix

### Chinese Version of the Inclusive Leadership Scale

#### Encouragement and Recognition to Employees

(1) In my work, the leaders actively ask my opinions and thoughts.(2) The leaders recognize the contribution of my efforts.(3) For my work, the leaders encourage me to come up with plans and ideas.(4) The leaders recognize our cooperation and exchanges across departments.(5) The leaders openly recognize the achievements of employees.

#### Respect and Fair Treatment for Employees

(6) The leaders treat us equally and always adhere to certain commonly recognized principles.(7) The leaders focus on fairness and justice when managing teams.(8) The leaders treat employees fairly.

#### Failure Tolerance

(9) When employees make mistakes, the leaders express emotional understanding and suggestions for improvement.(10) The leaders can rationally accommodate our mistakes.(11) When something went wrong, the leaders do not arbitrarily blame us without understanding the details.
